# Low-Income Families’ Direct Participation in Food-Systems Innovation to Promote Healthy Food Behaviors

**DOI:** 10.3390/nu15051271

**Published:** 2023-03-03

**Authors:** Aparna Katre, Brianna Raddatz

**Affiliations:** 1 College of Arts Humanities and Social Sciences, University of Minnesota Duluth, 1201 Ordean Court, Duluth, MN 55812, USA; 2 College of Education and Human Service Professions, University of Minnesota Duluth, 1201 Ordean Court, Duluth, MN 55812, USA

**Keywords:** food security, nutrition security, food deserts, community participation, low-income families, social innovation, food-systems innovation, meal kits, centering voices

## Abstract

Low-income families, especially those who reside in food deserts, face significant systemic barriers regarding their ability to access affordable and nutritious food. The food behaviors exhibited by low-income families are a reflection of the shortcomings of the built environment and conventional food system. Policy and public-health initiatives to improve food security have, thus far, failed to deliver interventions that simultaneously address multiple pillars of food security. Centering the voices of the marginalized and their place-based knowledge may result in the development of food-access solutions that are a much better fit for the population that they intend to serve. Community-based participatory research has emerged as a solution to better meet the needs of communities in food-systems innovation, but little is known about the extent to which direct participation improves nutritional outcomes. The purpose of this research is to answer the following question: how can food-access solutions authentically engage marginalized community members in food-system innovation, and if participation is related to changes in their food behaviors, how is it related? This action research project leveraged a mixed-methods approach to analyze nutritional outcomes and define the nature of participation for 25 low-income families who reside in a food desert. Our findings suggest that nutritional outcomes improve when major barriers to healthy food consumption are addressed, for example, time, education, and transportation. Furthermore, participation in social innovations can be characterized by the nature of involvement as either a producer or consumer, actively or inactively involved. We conclude that when marginalized communities are at the center of food-systems innovation, individuals self-select their level of participation, and when primary barriers are addressed, deeper participation in food-systems innovation is associated with positive changes in healthy food behaviors.

## 1. Introduction

Achieving food security is a persistent and severe issue in the United States—especially for low-income populations residing in food deserts. The United States Department of Agriculture (USDA) reported that 33.2% of low-income individuals in the United States lived in food deserts, and 10.2% of households were food insecure for at least a portion of time during 2021. Furthermore, 3.8% or 5.1 million households were highly food insecure [1]. Although historically in the United States it has been, food insecurity in food deserts should not be considered a solely geographical issue. Building supermarkets in low-income neighborhoods, connecting food-insecure residents to farmers’ markets, or other food-assistance programs may not improve dietary quality in the absence of other types of interventions [2]. Instead, food insecurity in food deserts should be addressed via multilevel approaches informed by an intersectional lens [3]. Interactions between social class, ethnicity, culture, economic status, and the food and built environment contribute to food behaviors displayed by low-income populations [2,4]. For example, family structure may influence how a household accesses and utilizes food. The challenges faced by Black, Indigenous, and People of Color (BIPOC) and single-parent families, who place a premium on time and convenience, are different from multigenerational and two-parent households, independent of socioeconomic status [2]. Ziso et al. [3] suggest that community-based participatory research may be a beneficial way to understand these interactions and center the voices of those most affected by food insecurity. However, even though knowledge about participatory research into food-systems innovation is expanding, as observed in the agroecology literature, studies about changes in food behaviors and nutritional outcomes, especially for low-income families, are lacking. In their systematic review evaluating community-based participatory research and interventions to improve food security, Doustmohammadian et al. [5] found just twelve studies, of which six were relevant for this research, i.e., they included a focus on nutrition and were conducted in poverty regions of developed countries. The interventions included store and shopping programs, fresh fruit and vegetable programs, nutritional education programs for mothers, and voucher/food-assistance programs. A deeper review of the six studies showed participation of marginalized voices in the intervention was limited to involvement in meetings and as research subjects. These studies did not explore the agency dimension of marginalized community members. Furthermore, the authors concluded that the study design in all cases was moderate/weak, and the nutritional interventions were ineffective for some food-access components.

Therefore, we set out to answer the question: how can food-access solutions authentically engage marginalized community members in food-system innovation, and if participation relates to changes in food behaviors, how does it do so? We begin by reviewing the literature on food behaviors of low-income families, food-security interventions and their outcomes, and community participation in food-systems innovation.

## 2. Food-and-Nutrition Security

### 2.1. Overview

The USDA defines food-and-nutrition security as “access by all people at all times to enough food for an active and healthy life” [6]. Food security ranges from high to very low, where the latter is characterized by several indicators of disrupted eating patterns and reduced caloric intake, https://www.ers.usda.gov/topics/food-nutrition-assistance/food-security-in-the-u-s/definitions-of-food-security/#ranges (accessed on 22 December 2022). In the United States, single-mother households and households with incomes below the poverty line have been identified as those with the highest rates of low and very-low food security [1,7]. These families often reside in food deserts that have severely limited availability of affordable and nutritious foods [8]. Although historically the USDA has prioritized unequal geographical access to food, causes of food insecurity, especially among low-income households, are systemic or the result of structural inequalities [8,9,10]. Food deserts have low availability of healthy food; median household income is at or below 185% of the federal poverty level (USD 51,338 for a family of four); a large share of the population (30% of households) lack access to a vehicle; and the distance to a supermarket is greater than a quarter of a mile. Fitzpatrick and Willis [4] found that the factors that characterize the residents of food deserts, for example, structural inequality, are correlated with diet-related chronic diseases.

However, despite limited household income contributing to poorer diet quality, some individuals can eat better for less—a concept known as nutrition resilience [2,3]. It is an individual or group’s ability to construct an affordable, appealing, and health-promoting diet in the face of potential inequities in the built food environment. Given the interplay between physical, economic, and social factors impacting an individual’s food decisions, Drewnowski and Kawachi [2] suggest research is needed into the promotion of culturally acceptable, healthy, and inexpensive foods. Exploring the diverse food procurement strategies and behaviors exhibited by low-income residents of food deserts provides insight into how interventions might adopt a multidimensional approach.

### 2.2. Low-Income Families’ Food Behaviors

The overemphasis of geographical access as a determinant of eating behavior has led to a new field of thought questioning whether physical distance is a true measure of access to healthy foods. The purchasing power of neighborhoods, in addition to household economic conditions, may be a much better indicator [2]. To come to these conclusions, Drewnowski and Kawachi [2] leveraged data from the Seattle Obesity Study (SOS II) and Kavli HUMAN Project (KHP) to paint a behavioral, economic, and cultural picture of diet quality and health in New York City. Belon et al. [11] conducted a mixed-methods study to establish how multiple environmental factors shape food decisions for the purpose of informing health policies and programs. Qualitative and quantitative analysis resulted in the emergence of four environmental themes: physical, sociocultural, economic, and political. The study found that, although people’s purchasing decisions are initially shaped by what is available within their built food environment, eating behaviors are nuanced by considerations of cost, sociocultural contexts, and policy [11]. A sense of belonging and understanding the complexities of perceptions of self and others are critical to low-income residents’ willingness to procure food at alternative venues such as mobile and farmers’ markets, food-giveaway events, and food shelves [12]. The Drewnowski and Kawachi [2] review portrays this concept using the phrase “build it and they may not come” (p. 194). For example, constructing a supermarket in a low-income area may not result in the declared manifest goal of improving the neighborhood’s nutritional status. Instead, sociocultural and economic interventions should be paired with transformations to the physical environment if improvements are to be made regarding the food-and-nutrition security of low-income households [2,11].

Low-income families appear to place a premium on time and convenience when it comes to food-purchasing decisions. Time scarcity, or limited time availability for cooking and shopping, is a significant barrier to the uptake of a health-promoting diet [11,12,13,14]. Increased time spent on food preparation and cooking has been linked to higher quality diets and health status [2,14]. These authors, however, suggest that low-income families experiencing time poverty due to unpredictable work schedules are more likely to eat out at fast-food restaurants and rely on packaged foods that are fast and easy to prepare. Monsivais et al. [14] hypothesized that an increase in time spent preparing, cooking, and cleaning up meals at home would be associated with healthier patterns of food consumption measured by an increase in fruit and vegetable consumption, decreased spending on food consumed outside of the home, and fewer visits to fast-food restaurants. The results of this study found a significant association between time and each of the named dependent variables, suggesting that cooking at home may be a prerequisite to consuming a healthy diet at the lowest possible cost. The exploration of individual-level and household factors may provide insight into low-income families’ food behaviors. For example, young working adults and single-parent families were found to be the most likely to eat away from home and the least likely to prepare home-cooked meals [2,13]. To support individuals who place a premium on time and convenience, quick-to-prepare, healthy, and affordable meals should be developed for, and advertised to, low-income families.

Household family structure appears to impact food-and-nutrition security [2], but little is known about the extent to which the nature of the family unit affects food choices. Household and individual-level food-insecurity data suggest that parents often shield their children from experiencing food insecurity [1]. Furthermore, working and single-parent families face significant challenges regarding their ability to shop for, prepare, and cook food for themselves and their children. In a study that sought to determine the ways in which working mothers allotted time for food provisioning, Jabs et al. [13] found that the helping roles of older children and contributing partners resulted in reduced feelings of time scarcity and stress regarding food preparation. Similarly, multi-generational households have demonstrated a more equitable distribution of food-preparation responsibilities alleviating pressure on the families’ younger, working adults [2]. Likewise, larger families, who are more likely to prepare and eat food at home, often consume more healthy diets [14]. Cultural practices and ethnicity also play a role in the food behaviors of low-income families. Sweeney et al. [15] leveraged thematic analysis to evaluate the food behaviors of African American and Hispanic low-income families for the purpose of developing a culturally appropriate meal-kit intervention. The study determined that both African American and Hispanic families had not yet considered meal kits as a way to overcome barriers (cost and time) to eating at home, despite the fact that both groups prioritized cooking and eating together as a family [15]. The review concludes that meal kits must be semi-tailored to address the taste and cultural preferences of the target audience if they are to improve the diet quality of families with low income [15]. For example, a meal kit prepared for a Hispanic family might include fruits, vegetables, and grains that are traditionally used in Hispanic cuisine. One can conclude that for nutritional resilience, the availability of culturally appropriate and acceptable foods that reduce cooking time and encourage family participation is critical to the uptake of a healthy diet among low-income families. 

The promotion of community-based solutions—such as local, sustainable food systems—may be a viable means to reduce food insecurity while fostering solidarity and connectedness within disadvantaged communities [11,16]. Food sovereignty, i.e., people’s ability to define their food systems for the purpose of ensuring their own livelihoods and ability to access culturally appropriate foods, may be considered a prerequisite for food security [17]. Food systems are often dominated by market economics. In areas with a higher concentration of racial and ethnic minorities, the overabundance of fast-food restaurants and convenience stores selling obesogenic food severely limits the food choices of low-income families [18]. Despite these conditions, some minority groups exhibit nutritional resilience. For example, Drewnowski and Kawachi [2] detail an analysis of diet quality in relation to cost that suggests that Mexican Americans are able to eat better for less. This draws attention to the need for educational interventions that teach low-income shoppers how to maximize their budgets while making the best food-related decisions [2]. To increase the health status of food-insecure populations and reduce disparities that exist along racial and socioeconomic lines, it is critical that access to healthy and culturally appropriate food improves. Ultimately, interventions that aim to reduce food insecurity must take into consideration the nuanced needs and cultural preferences related to the food choices of low-income populations.

### 2.3. Food-Security Interventions and Outcomes

A multitude of strategies and interventions have been employed by public health and governmental entities to combat food insecurity in the United States. Since a person’s food choices are directly affected by their food environment [3], interventions that look at food systems and facilitate multilevel interactions can result in favorable outcomes. Some examples include: multicomponent educational interventions, prolonged motivational campaigns, reduction of fruit and vegetable prices, discounted-produce markets, chef-run cooking demonstrations, shared recipes, community taste-testing events, and educational boxes [19].

Programs with educational components have been shown to reduce food insecurity among low-income populations. In one study, health-and-nutrition educational strategies, included providing healthy recipes, improving cooking skills, and informing individuals on the health risks associated with consuming processed and calorie-dense foods, were shown to improve diet-related outcomes [19]. In an effort to update the tools and technology available to the participants in a supplemental nutrition program for Women, Infants and Children (WIC) in the United States, a review was conducted to analyze the features of mobile phone apps available to the program’s participants. Apps that provided support while shopping and also featured nutrition-education modules were positively perceived by the program’s participants. These easy-to-use platforms were reported to be useful because of their ability to help save time spent shopping for WIC eligible items [20]. Personal nutrition education and programs culturally tailored to a specific group have also demonstrated effectiveness in improving the diet quality and food behaviors of low-income populations [21,22]. Finally, a longitudinal study [23] was conducted to evaluate the impact of school-based nutritional education on increasing children’s intake of fruits and vegetables. Strategies included increasing the frequency of cooking, the usage of nutritional labels in purchasing decisions, and the availability of fruits and vegetables. Changes to the home environment over a two-year period resulted in a significant decrease in total fat intake, in addition to an increased intake of fruits and vegetables for both children and parents [23].

However, educational interventions alone are insufficient, as cost perceptions and availability (related to transportation issues) may significantly impact consumption habits [3]. The Loopstra and Tarasuk [24] study examining the uptake and perceptions of community gardens, community kitchens, and food-box programs as a means to reduce food insecurity for low-income families in Canada showed low participation in any of the programs, even when the programs were in close proximity to the families’ residence. Primary causes included knowledge gaps of when, where, and how to participate, lack of information regarding its location or eligibility requirements, misalignment with family’s busy schedules, chronic health issues, and hesitancy in sharing communal spaces and having to work alongside strangers.

Place-based solutions capable of overcoming inequities in the conventional food system are opportunities to improve food security and reduce health disparities [4,25]. Food hubs, which aggregate, distribute, and sell locally or regionally sourced foods, may help to address lack of access to healthy food in certain low-income communities [25]. In fact, low-income residents perceive food hubs to be potentially more inclusive and aligned with their preferences, especially compared to farmers’ markets which, despite efforts to incorporate electronic benefits transfer (EBT) as a means to purchase goods, are considered to racialize, stratify, and exclude other low-income individuals [25,26]. Similar to the recommendations by Clapp et al. [17], these considerations underscore the importance of incorporating the voices of adults facing food insecurity in the detection of issues and the conception and design of food-access solutions within communities [11]. 

The preceding sections suggest that interventions embracing as many of the following characteristics as possible present the potential for optimal food-security outcomes for low-income families in food deserts: culturally appropriate, reduced cooking time, encouraging family participation, providing education, and being affordable.

### 2.4. Community Participation in Food Systems

Food security is widely understood to rest upon four pillars: availability, access, utilization, and stability. Clapp et al. [17] promote the incorporation of agency into policy and intervention frameworks developed to combat food insecurity. Sustainable food systems are respectful and empowering, where all people are able to make choices and exercise their voice in shaping the system. While measuring agency is challenging, Ziso et al. [3] emphasize the need to study it to understand the effectiveness of interventions.

Increasingly, communities are recognizing the importance of food citizenship, wherein people move beyond simply being food consumers to holding and developing capacities to actively engage in shaping the food system. Agency, defined as the capacity of individuals or groups to make their own decisions about what foods they eat and produce, and how that food is produced, processed, and distributed within food systems [27], is needed to shape food-system policies and governance. The HLPE [27] report elevates the need to promote the ability of food-system participants to exercise their agency and their right to food, as these are linked with improved food security [28] and developmental outcomes in general [29]. The report further recommends upholding governance structures to facilitate the protection of agency and food-and-nutrition security for all (p. 28). Designing public participation, including participation in food citizenship, is often conceptualized as a linear process with designated steps and execution methods. However, as Clark [30] shows through a case study, the approach has to be iterative, with several participation opportunities, so that local knowledge can be elevated to expert input. The study also recommends adopting a flat decision-making structure and taking the time to build consensus. As people’s collective-efficacy perception influences their participation in development initiatives [31], investing in building collective efficacy is imperative.

It is necessary to acknowledge the agency of the least advantaged in society in order to improve food security, but this topic is least understood. Marginalized communities may lack the agency to define their own place in, and relationships with, the food system, and approaches to strategically increase participation in low-income neighborhoods are not well understood. For example, Bornemann and Weiland [32] suggest that individuals can exercise agency by cooking at home and embracing values related to organic foods. However, prices, promotions, food types sold, and the proportion of heavily vs. less-processed foods are influenced by corporate concentration in the food-distribution and retail sectors, limiting low-income consumers’ ability to exercise agency [33]. At the community level, they can actively participate in local food initiatives such as food cooperatives, community-supported agriculture, and community gardens. However, such initiatives are often designed and deployed by nonprofit and public-sector organizations with their own agenda, but who act as trustees of the marginalized [34]. Marginalized communities who are most affected by food insecurity, when invited to participate, face information asymmetry and power differential affecting their ability to influence the solution. The level of beneficiary participation in developmental projects is associated with their perceptions of whether the projects will happen in reality, the success or failures of prior attempts, and their relationship with the agencies driving the change [34]. The author recommends a social contract that emphasizes the content of relationships between the various agencies and social groups, and rejects the idea of institutionalizing civil-society actors representing the marginalized.

Clark [30] and Prost [35] suggest that involving marginalized voices requires a commitment to social equity, and they call for investing in relationship building to make community participation accessible. Recognizing that not all of what is conveyed by marginalized communities will be integrated into food-systems design, Clark [30] recommends that trust developed through relationship building can be used to handle these situations. Therefore, scholars emphasize investing in relationship building and moving away from interventions focusing on skills development for the marginalized. However, food-democracy scholars, for example, Booth and Coveney [36], argue that to radically transform the food system, interventions need to improve the capacity of individuals and groups to act independently and make free food choices. This includes knowledge and skills related to growing your own food, home cooking, and challenging the rules and existing structures causing food insecurity, among others. Residents are more likely to engage in individual and collective action when they experience a sense of community, where there is hope for change and there is collective efficacy [36]. Laying out practical tools for increasing the participation of marginalized communities, these authors suggest beginning with active listening and challenging the beliefs of other food-system actors about the lack of participation, followed by mediation and negotiation to search for a shared solution. 

Social innovations and social enterprises play a key role in enabling individual and collective agency to transform food systems [37], including the agency of the disenfranchised and bringing about social inclusion at the community level [38]. Several examples of social innovation demonstrate that they establish social cohesion and socialization, helping fight the social exclusion of the marginalized. In such cases, social innovation enables the most excluded members to improve their circumstances, thereby developing and exercising collective agency. Fitzpatrick and Willis [39] suggest that collective agency is critical for allowing local communities to leverage place-based knowledge to shape their own food environments. Recognizing limited access to affordable and healthy foods as a place-based issue is central to developing place-based solutions which center on the local knowledge of community members most affected by the issue. Fernandez-Wulff [37] interviewed 104 individuals across an equal number of social innovations in the food system. They identified four dimensions of collective agency: consciousness, individual voluntary action, cooperative agency, and agency feedback loop. The social innovations in the study aimed to stimulate consciousness by improving information sharing and establishing new producer–consumer relationships, to increase control over food-related daily decisions. However, the finding of a lower level of consciousness among low-income communities suggests the need to create an environment where participants can engage authentically at their individual level of consciousness. Consciousness leads to individual voluntary action when social-innovation projects allow the heterogeneous participation of community members (best suited for the individual). Special consideration is needed of the disconnect between expert knowledge [37] or universal awareness and the lived experiences of low-income communities. This finding identifies a need for authentic, compassionate discourse that amplifies the voices of food-insecure populations for the purpose of transitioning to engaged action [37]. These individual actions can work as a stepping stone towards deeper engagement, progressing towards cooperative actions and collective agency. However, the transition in their study required “developing personal relationships with and among participants, designing activities for an active involvement, engaging participants on an individual level, and acknowledging and managing conflict as a natural part of the life of a group” (p. 10). While their study is significant in its scope and size, it lacks a special focus on strategies for mobilizing marginalized communities’ agency and mobilization outcomes. 

In conclusion, there is a growing interest in including disenfranchised communities in co-designing and decision-making for local food systems. Including community participation and the agency of marginalized groups as core dimensions of food security, Clapp et al. [17] recognize the challenges in defining and measuring it. While studies of food systems for community participation primarily focus on food-policy changes, this research attempts to analyze how food-systems innovation enables the participation of marginalized community members in food deserts and the resulting changes to their food behaviors. 

## 3. Materials and Methods

### 3.1. Research Setting

This action research project studies an evolving social enterprise, Food Forward, to uncover how marginalized community members engage, build capacities, and exercise agency in the social innovation of food systems, and to identify early indicators of food-behavior changes. The findings are situated and analyzed in the context of the collective-agency framework proposed by Fernandez-Wulff [37].

Food Forward is a nascent social enterprise whose mission is to provide low-income residents of Duluth, Minnesota’s Central Hillside neighborhood, with more equitable access to nutritious foods. This neighborhood is a food desert. Its residents face significant health disparities and, on average, have a life expectancy of more than ten years less than those residing in adjacent neighborhoods. According to the Community Health Needs Assessment (CHNA) conducted by Bridging Health Duluth in 2015 [40], 41.6% of Central Hillside adults reported feeling worried about running out of food. The root causes of food insecurity include limited income, lack of access to nutritious food, lack of transportation, and knowledge barriers [41]. Food Forward home delivers partially prepared meal kits to Central Hillside’s low-income residents once a week to help alleviate stress around food, including lack of knowledge of cooking and nutrition, financial stress, and transportation issues. The social enterprise leverages a participatory engagement framework that involves communities most impacted by food insecurity as consumers, and some, such as single moms receiving food stamps, in Food Forward’s design, development, and delivery of the service. For example, the participants made decisions about the choice of meal kits, striking a balance between familiarity, desirability, and nutritional value. Accessibility to healthy foods is a social determinant of health, but interventions targeted at this neighborhood in the past few decades are among those which have resulted in marginal improvements. Food Forward is supported by university and community partners.

### 3.2. Data Collection and Analysis

**Figure 1:**
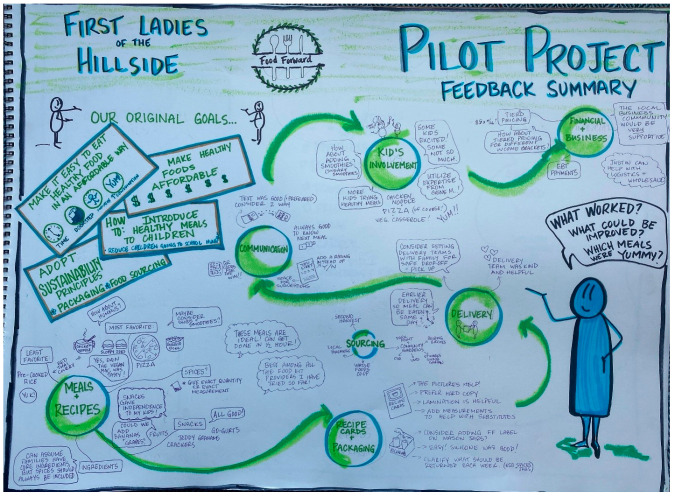
Graphic Recording of Focus Group 1.

**Figure 2:**
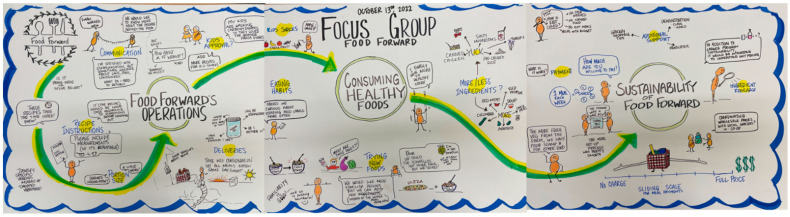
Graphic Recording of Focus Group 2.

Action research projects provide solutions to immediate problems and contribute to scientific knowledge and theory [42]. Exploratory studies such as this one benefit from qualitative methods [43,44]. Three kinds of data were collected. First, Food Forward provided the relevant documents: (a) social contracts established with various stakeholders, including its consumers, (b) its consumers’ demographics with no identifying information revealed, and (c) details about the meal kits and the delivery schedule. Second, Food Forward gathered anonymized survey-based meal-kit-specific consumer-feedback data. These data were used to review consumer satisfaction with the meal service (rating on a scale of 1 to 5) and to study open-ended consumer feedback. Third, all Food Forward consumers were invited to participate in two focus groups, lasting 60 to 90 min each, spaced over six months. The goals were to discuss their participation in the social enterprise and capture their food behaviors. Changes to their food behaviors were deciphered through new actions taken and support requested from the enterprise. An independent consultant captured graphic recordings of each focus group in real-time, enabling follow-through conversations with the consumers (see Figure 1 and Figure 2). Childcare, transportation, and snacks were provided to address barriers to participation in the focus group. An additional focus group was held with the First Ladies of the Hillside (see below for a description of this group) to capture their participation in production.

For each meal kit delivered, Food Forward provided the meal name, date, recipe card, ingredients, and the number of servings that were compiled into a cumulative “meal repository” for the research project. The survey data for meal kits were aggregated to develop descriptive statistics. Food Forward’s consumers in 2022 included 25 distinct low-income families on food stamps from Duluth’s Central Hillside. A maximum of 19 families and 56 servings were involved in any given week. Seven participants (families) represented one of the most vulnerable groups in the community regarding food-and-nutrition security—single mothers living primarily on welfare and in the city’s supportive housing, who are victims of generational trauma and have mental health issues. By design, this group of seven, the First Ladies of the Hillside, was involved in the conception of Food Forward and weekly service delivery. They had the greatest opportunities for participation as producers and consumers in social innovation. Over the course of the study, four remained consistently involved, whereas the participation of three diminished, eventually dropping off. Another seven families were actively involved as consumers, providing regular feedback to help develop the service and consuming healthy meals; one of these dropped out, due to changed life circumstances. The remaining eleven families were profiled as passive consumers, of whom three dropped off at various stages, and eight received the meals, providing feedback occasionally. Table 1 summarizes the family data, and Table 2 lists the meals delivered. Data about satisfaction with the meal, familiarity with the meal, involvement of children in making the meal, ease of preparing the meal, ease of following the directions and time taken to prepare the meals are summarized as the percentage of responses in various categories (see Table 3).

For qualitative data, we began by reviewing the social contracts with the First Ladies and with Food Forward’s consumers to identify participation opportunities and expectations. The facilitators at each table in the focus group made extensive notes of the discussions. These discussions and open-ended survey comments were compiled in a document. The coauthors separately studied the document, highlighting important fragments of texts as codes and marking them as actions demonstrating participation in the form of consciousness, voluntary actions, and barriers to participation. Through discussion and deliberations, the coauthors reached a consensus on the codes. Actions indicating consumers’ relationship with food and food-related behavioral changes conveyed as their needs, desires, and (dis)satisfaction with the meal-kit service were also highlighted. The codes were organized thematically into three groups representing different levels of participation, namely, passive consumers, active consumers not involved in the production, and active consumers involved in the production by design. Each group of codes provided a nuanced characterization of participation, demonstrating the consciousness of healthy foods and individual voluntary action. The food behaviors of typical consumers in the group were captured alongside. Preliminary propositions are developed by studying the differences in participation and food behaviors within and across the three groups.

## 4. Results

From its inception, the social innovation studied in this action research centers voices of the food-insecure, taking both producer and consumer roles while providing several participation avenues for other marginalized community members. The findings describe community participation in three primary categories, i.e., passive consumers, active consumers, and active consumers deeply involved in participation. We begin by presenting the changes to their food behaviors across the three groups.

### 4.1. Changes to Food Behaviors

Food Forward has a reasonably high retention rate of 72% over a year (Table 1), and while many meals were either unfamiliar or only a little familiar (43%), families reported high satisfaction with the meal (74%) (Table 3). Food Forward is achieving its goal to design meal kits so that meals can be prepared in less than 30 min, as indicated by 82% of the responses in this category (Table 3). Consumer feedback on early meal kits indicated dissatisfaction with the instructions to prepare meals. However, they took individual voluntary action, providing this feedback to help improve the instructions:

*“Last year I did some food subscription boxes that seem like the same idea and the recipes are easier to follow. I will also just attach a picture of one for an example. The way they are written make it simple, for example showing a ‘what you will need’ section on top is super helpful.”*

Over time, the satisfaction with instructions has improved significantly. Regarding involvement of children in preparing the meals, the high percentage of responses with little to no involvement (55%), indicates Food Forward has more work to do (Table 3). However, a majority of consumers (88%) found the meals easy to make (Table 3). These data suggest that Food Forward’s meal-kit model is headed in the desired direction to eliminate basic barriers of transportation and lack of familiarity with healthy meals, and, among others, to promote ease of cooking, contributing to simultaneously improving food and nutrition security. A few open-ended comments in the surveys support the above conclusions from the quantitative data:

*“I enjoy the fun of cooking with the kids,” “Our family have found the meals to be very easy and fast to prepare. Definitely helpful on a busy weeknight!, ” “I liked it, kids liked it, simple and fast. Beet hummus-no go,” “Great flavor and perfect for cold fall night. Easy meal and full of comfort.” “We are grateful to be a part of this pilot program. Thank you!” “This is a wonderful program”*

When the alternative is consuming less healthy foods, in an effort to not go hungry, families are choosing to give the service a try. However, when taste preferences are not met, families have reported reverting to usual eating habits to, oftentimes, appease children, as exemplified by these quotes:

*“I had to add meat for the kids,” “Kids weren’t a huge fan of the soup I think it was too healthy and out of their “comfort zone,” “they also associate it with being sick as that’s the only time we have eaten it before,” “It was a great meal idea, simple to follow easy to make,” “Kids refused it”*

The data also suggests that, at least for some children, receiving chopped vegetables and other ingredients in mason jars, and visually appealing recipe cards incite curiosity and the desire to participate in making the meal.

### 4.2. Passive Consumers

These are consumers who received the meal kit but did not participate in the focus groups or rarely provided feedback on the meal service. Many consumers signed up for the program so that their children could be oriented to eating healthily. Those who dropped off from the service cited a lack of their children’s interest in the meals. For instance, one consumer said, “(her child) does not like the food and so I want to withdraw.” When asked if they would continue for just the adults, the response was, “it is too much work to make two meals when [their child] isn’t interested.” When the meals did not meet the children’s preferences, and parents could not convince them, the service was no longer of value, and they chose to drop off. Other reasons involved personal life changes, wherein healthy eating was no longer a priority. The remaining seven passive consumers continued to participate by receiving weekly meals, thanking Food Forward during delivery. The only observable change to their healthy-eating practices was consuming one healthy meal a week. Thus, for passive consumers with low levels of participation in social innovation, changes, if any, to their relationship with healthy foods, are minor. 

### 4.3. Active Consumers

This group consisted of consumers who took a more active role in providing feedback to help improve the service. Feedback was gathered during meal-kit deliveries, through weekly surveys specific to the meal for the week, and through bi-annual focus groups. These opportunities served as places where consumers took advantage of a direct line of communication with Food Forward. Only one consumer requested to stop receiving the meal kits. This consumer was involved in voluntary action for meal-kit deliveries on at least five occasions and referred other needy consumers to the program, but had to drop out, due to changes to their personal life circumstances.

Many active consumers share social networks with the First Ladies, and participating in Food Forward provided opportunities to connect. One consumer described their desire for connection as, “humanize the project. Highlight the work of the First Ladies, put faces to the force behind Food Forward.” These consumers had provided feedback on several occasions to improve Food Forward’s operations. For example,

*“provide serving sizes on recipe cards when quantities provided are more than needed,” “placing the return labels so that they are more visible,” “we’re an early to bed household. It’d be preferable for our family to be able to cook the meal the next day,” “make past recipes searchable on a website,” and “I really appreciated the reusable container component. Unfortunately, we use the same(* *ish* *) jars in our household and I’m sure they got used interchangeably.”*

The feedback resulted in improvements such as adding Food Forward labels to the mason jars, moving the delivery time to earlier in the day and providing single-serving sizes on the recipe cards.

Active consumers shared information demonstrating changes to their relationship with food. For example, *“my diabetic son is getting exposed to new foods,” “knowing that the vegetables are fresh coming from a local farm makes me comfortable, we are avoiding the pesticides,” “it changes up our dinner routine. It makes us more open to trying new foods and some healthy foods,” and “the meals are expanding our horizons because of the new meal each time.”*

These consumers demanded more support from Food Forward to transition into eating more healthily (see Figure 1 and Figure 2). For example, one consumer requested alternatives, tips, and instructions, so they could repeat the meal all by themselves; another asked for substitutes they could use in a meal and demanded recipes for sauces and curry paste prepared by Food Forward. 

### 4.4. Active Consumers Deeply Involved in Production

This group constitutes the First Ladies of the Hillside. As both producers and consumers of Food Forward’s service, First Ladies are at the core of this investigation. Engagement, curiosity, viewing the opportunity to support their families and community in promoting positive health outcomes, and consistently showing up, are some emerging themes in this research, despite several barriers to participation. Food Forward addressed basic barriers to their participation in production by providing free childcare and transportation to the production site, and by compensating them. The study revealed that their participation in production and as consumers are correlated. These findings are presented below in two groups, one for the group that stayed consistently engaged, and another with wavering participation.

Inconsistent participation: Three of the seven First Ladies demonstrated inconsistent participation throughout the study. For the purpose of this study, they are referred to as participants A, B, and C. Their choice to receive meals mirrored their ability to stay involved in the production. Mental health issues attributed to past trauma were cited as the primary reason for inconsistent participation as a consumer and producer. Participant A first discontinued participation in production, lacking any communication with Food Forward. Shortly after, they were dropped from the consumer list. Participant B initially stayed engaged with production but wavered as a consumer, citing the reason, “I have a lot going on at home, do not have time to cook the meal.” They preferred to resort to unhealthy processed food, even though free healthy food was on the doorstep and could be cooked in less than 30 min. Eventually, participant B also dropped off from the production side. Participant C, like A, stopped being involved in the production first. Some of the First Ladies were able to leverage their social network to break through communication barriers and uncover the causes, which were once again related to mental health and trauma. These findings suggest that even when other barriers are eliminated, mental health and trauma continue to disrupt their ability to consume healthy foods.

Consistent participation: The remaining four First Ladies received 27 meals over the study duration. Since they were core for production, deep insights were gained about factors influencing their food choices and changes in food behaviors. Most conveyed that they didn’t necessarily use all the contents of the meal and would drop some vegetables, for example. They reported trying new meals, such as beet hummus, squash risotto, and yellow curry, simply due to their involvement in preparing the meals and assembling the meal kits. They have even reported repeating the new meals mentioned above. Some have reported developing new cooking habits, such as cooking the meal using Food Forward’s meal kits after a full day of work, whereas, in the past, they would have resorted to processed, ready-to-eat meals. Unlike some passive consumers, the women in this group would continue to cook the meal and remain a consumer even if their children didn’t eat it. Upon probing, one of them said, “we can set an example, and eventually [the child] might be interested in eating it”. Clearly, initiative and individual voluntary actions were more abundant in this group, compared to other groups.

## 5. Discussion

The findings reinforce the fact that low-income families face many barriers that impact their ability to consume a health-promoting diet. It is critically important that efforts to reduce these barriers are anchored in the knowledge possessed by marginalized groups. This study reinforces Vincent’s [34] suggestion of social contracts as a mechanism to enable the direct participation of marginalized groups instead of trustees representing their interests. As seen in this study, the contradiction in the literature of investing in relationship building [30,35] vs. skills development [36] can be resolved through a model that directly involves marginalized communities. Their shared identities and social groups provide the necessary relationships and trust, whereas codesigning the social innovation and involvement in production helps skill building. As this study demonstrates, there are many opportunities in which expert knowledge can be blended with traditional knowledge and the lived experiences of marginalized communities to raise their consciousness of healthy foods and stimulate individual voluntary action. The propositions introduced at the end of this discussion represent a culmination of the literature, theory, and findings from the social innovation studied. We begin by discussing the short-term outcomes of social innovation, followed by approaches to enabling multiple levels of authentic engagement and the specific outcomes observed.

### 5.1. Changes to Food Behaviors

Interventions for low-income families in food deserts addressing only one dimension of the food-access issue help develop healthy-eating awareness, but rarely result in sustained changes to food behaviors [2,9,11,15], as other barriers to healthy eating persist. Meal kits are boxes of pre-portioned ingredients for a set number of meal servings, along with step-by-step instructions for cooking the meal at home. Home-delivered, partially prepared meal kits address several barriers to home cooking, which, as per Monsivais et al. [14], is a prerequisite to consuming a healthy diet. Meal kits can eliminate barriers of cost, time to shop for ingredients, food storage, cooking time, and food-preparation knowledge. Due to their multidimensional approach, home-delivered meal kits can create a favorable food environment and, as suggested by Ziso, Chun [3], can influence an individual’s dietary habits. Even though the meal-kit market is large (USD 7.64 billion for the U.S. in 2022) and growing, there are a few issues: they serve individuals with higher incomes, and of the 18% estimated population who are buying meal kits, the frequency of usage is low, with the vast majority ordering only a few times a year [45]. Early studies exploring changes to food habits as a result of meal kits are promising [46,47,48]. To the best of our knowledge, Carman et al. [48] is the only study exploring the acceptability of meal kits among low-income families. The results of our study support their findings, wherein Food Forward’s consumers also demonstrated a high rate of satisfaction and retention in the program for an entire year, especially when the only incentive was free meals. We offer two explanations for a higher acceptance of Food Forward’s social innovation among food-insecure families, compared with those in the Loopstra and Tarasuk [24] study. First, as suggested by several scholars, for example [11,13,14], the innovation prioritized eliminating cooking-time barriers by providing chopped vegetables and premade sauces and home delivery, keeping the meal preparation time under 30 min. Second, providing authentic engagement avenues [37] where consumers could provide feedback and see prompt improvements could have motivated them to continue with the service. 

Consumers requesting redesigned recipe cards indicated their desire to learn to cook new meals, and requests for full recipes and substitute ingredients demonstrated their desire to make dietary changes toward healthy foods. The study confirms that partially prepared meal kits with the proper support (home delivery, chopped vegetables, and easy instructions to cook) can encourage healthy food behaviors.

### 5.2. Participation in Social Innovation and Changes to Food Behaviors

Prost [35] suggests the need to make participation in food-system-innovation accessible, especially for marginalized voices. Food Forward is designed to accommodate varying levels of participation at the discretion of the consumers (primarily low-income families living on food stamps), thus providing authentic engagement opportunities, as Fernandez-Wulff [37] suggested. This section discusses the association between varying participation levels and food-behavior changes, to arrive at propositions.

Individuals’ perceptions of their food environment may impact their food behaviors [3], but deeper participation in food-systems innovation is associated with increased awareness of healthy food behaviors. Contrary to the current literature, which largely focuses on participatory research methods (for example, Ziso, et al. [3]), this work centers on the voices of the marginalized via direct participation in developing the social enterprise model. This highly engaged, multi-level method of altering the food environment in Duluth’s Central Hillside has demonstrated that those most deeply involved have increased knowledge and awareness of healthy food behaviors. Loopstra and Tarasuk [24] conclude that the uptake of food-assistance programs by low-income families is directly related to their perceptions of the program. Specifically, is the program accessible, and does it fit into the family’s lifestyle and behaviors? Food Forward consumers’ self-determined participation is authentic, and creates an opportunity for low-income families to formulate perceptions of improving their food system based on first-hand experiences.

Fernandez-Wulff [37] recognizes the nonlinearity of individual participation and collective expression in democratized food systems. This conclusion reinforces the need to validate the barriers that impact low-income individuals’ ability to engage in social innovations. Food Forward eliminated basic barriers by providing multimodal-feedback opportunities. For example, conversing with the delivery personnel, meal-specific paper-based and online feedback forms, and the invitation to participate in focus groups. Additional measures for focus groups included providing meals, childcare, and transportation services. The same measures were necessary to enable participation for consumers who were also involved in the day-to-day production and service delivery.

The data suggest that as individuals deepen their level of participation in the food system innovation, they appear to take increasing levels of voluntary action toward improving their dietary behaviors. For example, passive consumer participation has been limited to receiving meals. Due to limited feedback and participation in focus groups, little is known about changes in healthy food behaviors other than weekly consumption of meals and retention as a consumer. Active consumers have taken voluntary action in providing feedback, deepening their social networks, and involving their children in the preparation of meals. Data suggests that active consumers experience social inclusion and a sense of community. Thus, meal kits foster solidarity and connection with and among disadvantaged communities, and, as suggested by Belon et al. [11] and Myers and Painter [16], these are important factors in fostering food security. These individuals are exercising agency (as suggested by Šimundža [38] and Booth and Coveney [36]) to improve their relationship with healthy foods. Finally, those involved in production have demonstrated an increase in their knowledge of preparing healthy meals and also their willingness to try new foods.

The above discussion suggests that when marginalized communities are at the center of food-systems innovation:

**Proposition** **1a:** Marginalized voices self-select their level of participation.

Further, when primary barriers to participation are addressed:

**Proposition** **1b:** Deeper participation in food-systems innovation is associated with positive changes in healthy food behaviors.

However, some of Food Forward’s consumers involved in production demonstrated inconsistent participation, citing mental health and trauma as the primary reasons. Despite an option to stay enrolled in the service, these individuals chose to opt out of the program, both as consumers and producers. This finding highlights the need for additional support for low-income individuals who experience mental health issues and trauma, if they are to increase their participation in food-systems innovation and, therefore, their healthy eating behaviors. As a result, we propose that: 

**Proposition** **2:** Opportunities for deep participation alone are insufficient to produce healthy food behaviors. An individual’s worsened mental health:

**Proposition** **2a:** is associated with decreased participation in food-systems innovation, and

**Proposition** **2b:** interrupts changes in healthy food behaviors

## 6. Conclusions

The importance of involving marginalized communities in shaping their food systems to increase healthy eating outcomes cannot be overstated. This research suggests that limited access to affordable and healthy foods is a place-based issue that requires a solution informed by local knowledge. Marginalized and low-income groups face an array of systemic barriers but, when given the opportunity to participate in food-systems innovation, demonstrate positive changes in healthy food behaviors. Efforts to combat food insecurity in low-income populations would be well served by adopting an integrated approach to eliminating multiple barriers. More important, though, is adopting an agency-enabling perspective to developing intervention programming and policy framework. This research emphasizes the value of starting from the social groups of marginalized communities where strong relationships and trust exist, and facilitating the growth of their social networks and skills, in turn increasing their capacity to affect change. Preliminary propositions can guide future research on studying the effects of direct participation of food-insecure communities on their dietary outcomes.

This research is limited to a single case which is still in its early stages of innovation, and involves a limited number of participants. The analyses used mixed qualitative and quantitative data to examine emerging themes and develop propositions. However, the research did not focus on theoretical saturation, and the statistical analyses were limited to descriptive and summary statistics characterizing retention rates and consumer satisfaction. Therefore, the propositions should be considered preliminary, and used as guidance for future research design.

## Figures and Tables

**Table 1:** Family Characteristics, Participation, and Retention by Nature of Participation.

Nature of Participation	Family Demographics	Percentage of Participating Families (*n* = 25)	Total Retention Rate
Passive consumers.	Low-income. Utilizing food stamps.	44% (*n* = 11)	72.7%
Active consumers, uninvolved in production.	Low-income. Utilizing food stamps.	28% (*n* = 7)	85.7%
Active consumers, involved in production	Single-mother household. Welfare and supportive housing. Mental health issues. Generational trauma.	28% (*n* = 7)	57.1%

**Table 2:** Food Forward Meal Kits Delivered in 2022.

Spring 2022 Meal Kits	Fall 2022 Meal Kits
Broccoli Chicken Stir Fry	Yellow Curry Noodles with Meat and Vegetables
Chicken Tacos	Basil Pesto Pizza
Spaghetti	Vegetarian Shepherd’s Pie
One-Pot Chicken Dumpling Soup	Vegetable Risotto with Bacon
Red Thai Curry	Cheesy Group Beef and Cauliflower Casserole
Veggie Mac and Cheese	Rice with Chicken, Summer Squash, and Green Beans
Vegetable Pizza	Stewed Beef and Tomatoes
Burrito Bowl	Seasonal Veggies and White Bean Stew
	Brown Butter and Sage Pasta with Oven Roasted Potatoes
	Seasonal Veggies and Beef Stew

**Table 3:** Summary of Weekly Feedback Survey Results.

Category	Very/Super	A Little/Somewhat	Not at All
Satisfaction With the Meal.	75.5%	20.4%	4.1%
Level of Familiarity with the Meal.	57.1%	30.6%	12.2%
Level of Child Involvement in Preparing the Meal	35.6%	28.9%	35.6%
**Category**	**Very Easy/Easy**	**Somewhat Easy**	**Difficult/Very Difficult**
Ease of Preparing the Meal.	60%	13.3%	26.6%
Ease of Understanding Recipe Directions	75.5%	12.2%	12.2%
**Category**	**30 min**	**30 min or More**	
Meal Preparation Time.	83.7%	16.3%	

## Data Availability

The data presented in this study are available on request from the corresponding author. The data are not publicly available due to privacy reasons.
